# One-Pot Synthesis of Pd Nanoparticles Supported on Carbide-Derived Carbon for Oxygen Reduction Reaction

**DOI:** 10.3390/nano14120994

**Published:** 2024-06-07

**Authors:** Madis Lüsi, Heiki Erikson, Maike Käärik, Helle-Mai Piirsoo, Jaan Aruväli, Arvo Kikas, Vambola Kisand, Jaan Leis, Kaupo Kukli, Kaido Tammeveski

**Affiliations:** 1 Institute of Chemistry, University of Tartu, Ravila 14a, 50411 Tartu, Estonia; 2 Institute of Physics, University of Tartu, W. Ostwald Str. 1, 50411 Tartu, Estonia; 3 Institute of Ecology and Earth Sciences, University of Tartu, Vanemuise 46, 51014 Tartu, Estonia

**Keywords:** oxygen reduction reaction, palladium catalyst, Pd nanoparticles, carbide-derived carbon, electrocatalysis

## Abstract

We explored two methods for synthesizing Pd nanoparticles using three different carbide-derived carbon (CDC) support materials, one of which was nitrogen-doped. These materials were studied for oxygen reduction reaction (ORR) in 0.1 M KOH solution, and the resulting CDC/Pd catalysts were characterized using TEM, XRD, and XPS. The citrate method and the polyol method using polyvinylpyrrolidone (PVP) as a capping agent were employed to elucidate the impact of the support material on the final catalyst. The N-doping of the CDC material resulted in smaller Pd nanoparticles, but only in the case of the citrate method. This suggests that the influence of support is weaker when using the polyol method. The citrate method with CDC1, which is predominantly microporous, led to a higher degree of agglomeration and formation of larger particles in comparison to supports, which possessed a higher degree of mesoporosity. We achieved smaller Pd particle sizes using citrate and NaBH_4_ compared to the ethylene glycol PVP method. Pd deposited on CDC2 and CDC3 supports showed similar specific activity (SA), suggesting that the N-doping did not significantly influence the ORR process. The highest SA value was observed for CDC1/Pd_Cit, which could be attributed to the formation of larger Pd particles and agglomerates.

## 1. Introduction

Pd has been researched as a substitute for Pt as the oxygen reduction reaction (ORR) catalyst for the fuel cell cathode side due to its structural similarity and greater availability in the Earth’s crust [1,2,3,4]. However, the comparison of Pd with Pt is only competitive in alkaline conditions [5,6,7]. Kim et al. and Arriaga et al. noted higher electrocatalytic ORR activity in alkaline media on Pd/graphene in comparison to Pt/graphene [5,7].

There are three routes for improving the utilization of noble metals in the ORR catalyst material: reduction of loading of noble metal, nano-alloying, and improving dispersion and stability through substrate interactions [1,2,3,4]. This work focuses on the third category, which employs carbide-derived carbon (CDC) materials to support Pd particles.

Pd nanoparticle (PdNP) sizes and shapes are generally limited to a small pool of suitable sizes for the ORR. Jiang et. al. studied the ORR in alkaline media while varying Pd particle sizes between 3 and 16.7 nm, with mass activity showing a volcano-type plot with a maximum of around 5 nm and the specific activity (SA) increasing with Pd particle size [8]. The SA effects have also been noted on thin Pd films [9]. Furthermore, different facets of Pd possess varied ORR activity, and as such, shape-controlled Pd particles dominated by a specific facet exhibit different ORR activity, with the benefiting shape being cuboid [10,11,12,13,14,15]. The particle shape effect observed in acidic conditions is conclusive; however, in alkaline media, Shao and co-workers observed no difference in electrocatalytic activity for their cubic PdNPs compared to spherical ones [16]. This is also supported by a single-crystal study carried out by Hoshi and co-workers on Pd monocrystals in alkaline media, as it was shown that the ORR activity increases in the order Pd (110) < Pd (100) < Pd (111) [17]. However, the differences in the electrocatalytic ORR activity are not as drastic as in HClO_4_ [18].

Various synthesis methods for PdNP preparation have been studied, which include methods such as microbial and other green methods using plant extracts; however, these methods are somewhat impractical and rarely produce suitable particle sizes for ORR application [19,20,21,22,23,24,25]. The noble metal particle size must be below 5 nm to obtain any reasonable mass activity for ORR. Still, the number of synthesis methods for achieving these small particles is limited. Methods such as water-in-oil microemulsion require a lot of solvents and are generally not green approaches for particle synthesis; however, these are suitable to achieve the desired particle size of 5 nm and less [26,27]. Reduction of Pd precursors with alcohol usually leads to larger particles except for anhydrous methanol [28,29,30], but reduction with ethanol produces particles of 3–12 nm [24]. However, variation of ethanol content with polyvinylalcohol capping agent could lead to better particle size control [31]. There are two suitable methods of PdNP synthesis based on NaBH_4_ reduction in the presence of citrate ions and reduction with H_2_ at elevated temperature [32]. Ethylene glycol (EG) has been used as a reducing agent for PdNP synthesis; however, typically, larger particles are formed. With the addition of polyvinylpyrrolidone (PVP), Pd particles of 4–14 nm have been obtained [33]. However, the addition of hydroxide has been shown to produce a suitable Pd particle size [34], and this method was used in this work for comparison.

Since the substrate can influence the particle size during the synthesis, one-pot synthesizes can be challenging to reproduce [35,36,37]. It has been shown that the presence of nitrogen groups on the support can lead to smaller PdNPs compared to the non-doped counterpart, which usually leads to a better dispersion of particles and improved catalyst mass activity. According to a study by Perini et al., the use of mesoporous carbon and nitrogen doping of the carbon support resulted in a decrease in particle size from 5–6 to 2–3 nm without the need for surfactants [38]. Similarly, a reduction in particle size was observed by depositing the Pd catalyst on graphene and N-doped graphene using plasma synthesis, as it decreased particle size from 2.8–3.2 to 2.6–2.9 nm [37]. This effect has also been noted using a PtM (Pd, Fe, Ni) catalyst, as smaller nanoparticles were observed for all alloys on N-doped carbon black compared to regular undoped carbon black [39]. Another benefit of N-doping has been noted by Perini et al. and Zhang et al., showing an increase in stability with N-doped support material [38,40], with contradicting results shown for HOPG, in which case N-doping lowered stability [41]. Various materials have been used to support metal nanoparticles for ORR, including covalent organic frameworks and oxides, which can benefit catalyst stability [42,43].

Two one-pot synthesis methods were used: one with EG as the reducing agent and PVP as the capping agent, and the other with NaBH_4_ as the reducing agent and citrate as the capping agent. Three different CDC materials were chosen as support materials, of which CDC3 was nitrogen-doped. EG synthesis was only used for CDC2 and CDC3 since these performed best using the citrate method. The prepared catalyst materials were characterized by TEM, XPS, MP-AES, and electrochemically using a rotating disc electrode.

## 2. Materials and Methods

Three carbide-derived carbon (CDC) support materials were employed in this work. The CDC materials were synthesized by chlorine treatment at a high temperature. The starting carbides used for the CDC preparation are TiC for CDC1 [44], B_4_C for CDC2 [45], and TiCN for CDC3 [46]. The porosities of these materials are provided in Table S1 [45], and corresponding pore-size distribution (PSD) graphs are shown in Figure S1. These CDC materials were ball-milled according to a previous procedure using 4 mL of ethanol, 200 mg of CDC, and 400 rpm for four cycles of 30 min and 5 min cool-down periods with 0.5 mm balls [47].

CDC/Pd_Cit materials were prepared using a simple one-pot synthesis. A certain amount of PdCl_2_ (Sigma Aldrich, St. Louis, MO, USA) was dissolved in water (Millipore, Inc., Burlington, MA, USA) and HCl (Sigma Aldrich), to which sodium citrate (Sigma Aldrich) and CDC support were added. PdCl_4_^2−^ and citrate concentrations were 0.25 mM, in which 80 mg of a CDC material was dispersed. For all catalysts, the nominal 20 wt% Pd loading was used. During stirring, 22.3 mL of freshly prepared ice-cold 0.1 M NaBH_4_ (Aldrich) was added, followed by stirring for 30 min. After stirring, KOH pellets were added to remove citrate, and the material was filtered and dried in an oven overnight at 60 °C. Catalysts are named CDCx/Pd_Cit with x representing the used CDC.

CDC/Pd_EG materials were synthesized by dissolving 20.9 mg of PVP (MW = 8000) and 20.9 mg of PdCl_2_ in 25 mL of EG, where 50 mg of CDC had already been dispersed for acidification; 21 μL of conc. HCl solution was used in the case of CDC2 and conc. H_2_SO_4_ in the case of CDC3. After that, 7.6 mL of EG in which KOH was dissolved was added to reach 0.3 M final hydroxide concentration. The reaction bath was heated to 190 °C for 1 h. After that, EG was evaporated at 200 °C in a N_2_ atmosphere. Carbon material was re-dispersed in 3 M KOH to remove remnants of PVP and centrifuged three times, followed by filtering and drying overnight at 60 °C. Pd loading for the catalysts was aimed at 20 wt%. Catalysts are named CDCx/Pd_EG, with x designating the used CDC.

CDC/Pd catalyst materials were dispersed in isopropanol (99.8% Honeywell)/Nafion (5%, Aldrich)/water mixture at a volume ratio of 0.975:0.025:9, of which 6 μL was pipetted onto a glassy carbon (GC) disk electrode (d = 5 mm, Origalys, Rillieux-la-Pape, France) rotating at 700 rpm and dried in N_2_ flow. For comparison, a commercial Pd/C catalyst (20 wt% Premetek, Cherry Hill, NJ, USA) was employed in this work.

For X-ray photoelectron spectroscopy (XPS) measurements, the samples were drop-casted onto GC plates and measured using Scienta SES-100 electron energy analyzer (at 200 eV pass energy, Gammadata Scienta AB, Uppsala, Sweden) using Mg Kα radiation (incident energy 1256.6 eV) from twin anode X-ray source XR3E2 (Thermo Electron Corporation, East Grinstead, UK) with the total energy resolution of about 0.7 eV. The incident angle was 45°, and the take-off angle was 0° relative to the sample normal. The pressure in the analysis chamber was about 5 × 10^−10^ Torr. Spectra were calibrated using Au 4f photoelectron peaks. CasaXPS software (2.3.22PR1.0) was used for data analysis. Before fitting, the X-ray satellite was subtracted. For curve fitting, we used the combination of Gaussian and Lorentzian line shapes (GL30) for line shape and for the background correction the combination of linear and Shirley backgrounds. For sp^2^-C the asymmetry was added by convolution with a Doniach–Sunjic line shape. Scanning transmission electron microscopy (STEM) images were obtained by imaging samples drop-casted onto a lacey carbon-coated copper grid using a transmission electron microscope Titan Themis 200 (FEI) (Thermo Fisher Scientific, Waltham, MA, USA) operating at 200 kV using HAADF and BF detectors. Imaging was carried out in scanning mode and Gatan Digital Micrograph software (2.12.1785.0) was used for image analysis. The same operating voltage and spot size were implemented for elemental mapping with the SuperX (Bruker, Billerica, MA, USA) energy-dispersive X-ray spectroscopy (EDX) system. The signal was acquired for 10 min.

For microwave plasma atomic emission spectroscopy (MP-AES) analysis, the Anton Par Multiwave PRO microwave digestion system with NXF100 digestion vessel was used to dissolve 10 mg of catalyst material in 8 mL aqua regia. The metal content of the catalysts was determined using Agilent MP-AES 4210 (Agilent, Santa Clara, CA, USA). The crystallographic structure of catalysts was studied by X-ray diffraction (XRD) using a Bruker D8 Advance diffractometer (Bruker, Billerica, MA, USA) with Cu Kα radiation and 1D-detector (LynxEye XE-T). Profile fitting was carried out using Bruker TOPAS 6.

A standard three-electrode system was used for electrochemical measurements. The following working, counter, and reference electrodes were used: GC electrode covered with a catalyst layer, Pt-wire, and Pt wire in an electrolyte solution bubbled with continuous H_2_ flow and separated from the working electrode compartment with Luggin capillary. Experiments were carried out in 0.1 M KOH solution (p.a., Aldrich) and saturated with Ar (99.999%, Linde Gas, Dublin, Ireland), O_2_ (99.999%, Linde Gas) or CO (99.97%, Linde Gas). For the rotating disk electrode (RDE) measurements, a rotator and speed control unit (OrigaLys ElectroChem, SAS, Rillieux-la-Pape, France) were used, and electrode potential was controlled with PGSTAT128N potentiostat/galvanostat (Metrohm Autolab, Utrecht, The Netherlands). The electrochemistry data were collected using GPES software. Cyclic voltammograms (CVs) were measured between 0.1 and 1.4 V vs. RHE at a scan rate of 50 mV s^−1^. The electrodes were conditioned between 0.1 and 0.8 V for 10 cycles at 50 mV s^−1^. CO stripping was carried out at 20 mV s^−1^ between 0.1 and 1 V. The ORR polarization curves were measured between 0.1 and 1.1 V vs. RHE at a potential scan rate (*v*) of 10 mV s^−1^. The electrode rotation rate (*ω*) was varied between 360 and 4600 rpm. Electrochemical measurements were made at room temperature (23 ± 1 °C).

## 3. Results and Discussion

### 3.1. TEM and MP-AES Studies

**Figure 1:**
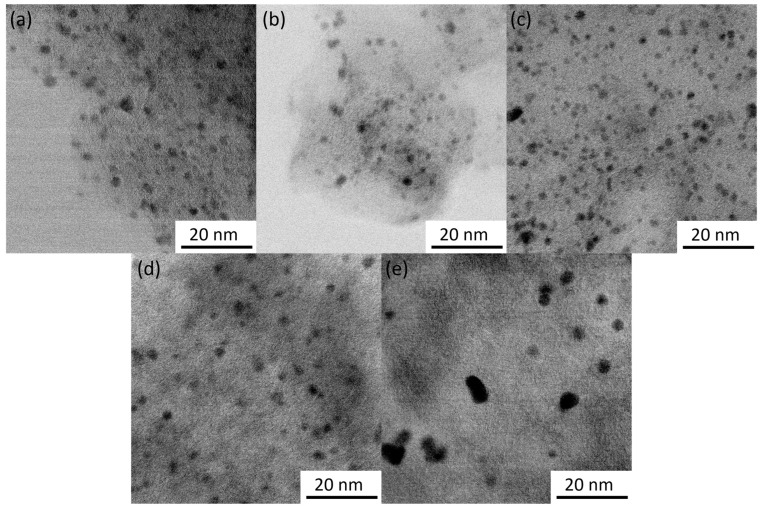
STEM images of CDCx/Pd catalysts: (**a**) CDC1/Pd_Cit, (**b**) CDC2/Pd_Cit, (**c**) CDC3/Pd_Cit, (**d**) CDC2/Pd_EG and (**e**) CDC3/Pd_EG. Scale bar 20 nm.

**Figure 2:**
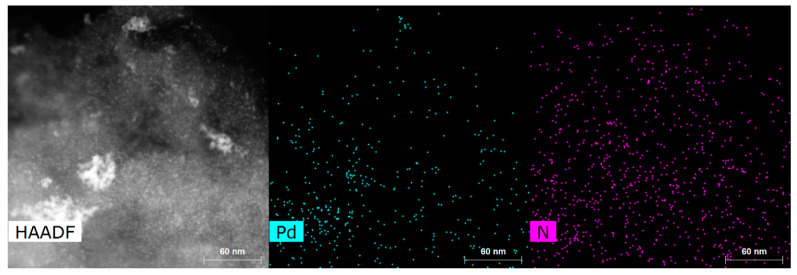
STEM image and elemental mapping of CDC3/Pd_Cit.

The average lattice spacing of 2.27 Å with standard variation of 0.06 Å was determined from STEM images of the particles. Examples of lattice fringes are shown in Figure S2. This distance can be attributed to the (111) planes of face-centered cubic Pd (2.227 Å) [48], which is also supported by XRD data (see next section). Particle sizes were counted from the TEM images and are provided in Table 1 with particle size distribution histograms in Figure S3. The citrate method resulted in smaller Pd particles in comparison to the EG/PVP method for both CDC2 and CDC3 substrates. Pd nanoparticles deposited on CDC1 showed similar particle sizes. However, a higher degree of agglomeration was observed. Generally, it has been shown that the presence of nitrogen functional groups in the carbon material leads to smaller PdNPs in the one-pot synthesis methods [38,39,49]; however, in the case of the EG/PVP method used in this work, smaller Pd particles were obtained on the CDC2 support, while the citrate method gives a conventional result. Furthermore, another driving factor for the deposition of metal nanoparticles is the defectiveness of the support material, as shown by Asanova et al., where significantly better nanoparticle dispersion and smaller size were achieved on a support with more defects [50]; this has also been observed in our electrodeposition work [36]. Figure 1e shows that the PdNPs for CDC3/Pd_EG are not spherical compared to the CDC2/Pd_EG material (Figure 1d), suggesting that the support has played an essential role in shaping these Pd particles. Figure 2 demonstrates the N-doping of the CDC3 material, showing well-distributed nitrogen species across the entire material.

Pd content was determined by MP-AES (Table 1), and we can observe that for the one-pot synthesis of Pd particles, the support material has played a role in Pd metal uptake with microporous CDC1 having lower Pd loading compared to mesoporous CDC2 and CDC3.

### 3.2. XRD and XPS Analysis

**Figure 3:**
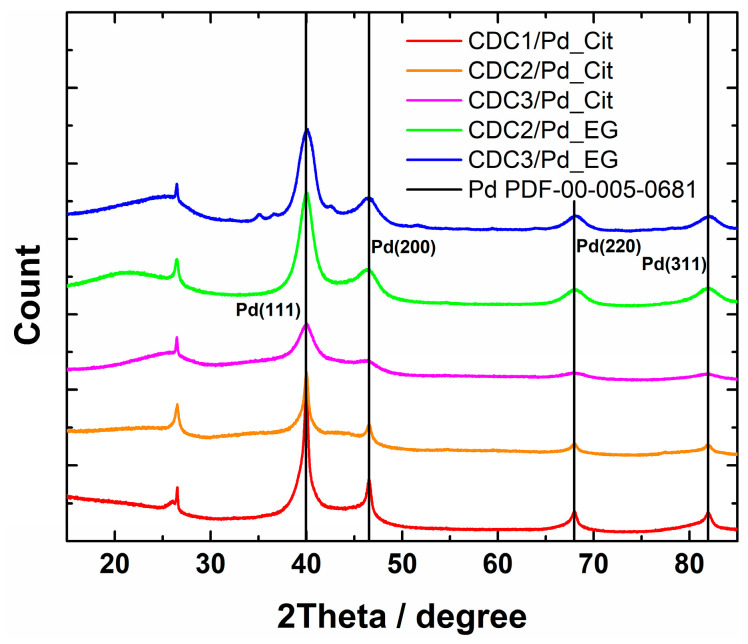
XRD patterns of CDCx/Pd materials.

**Figure 4:**
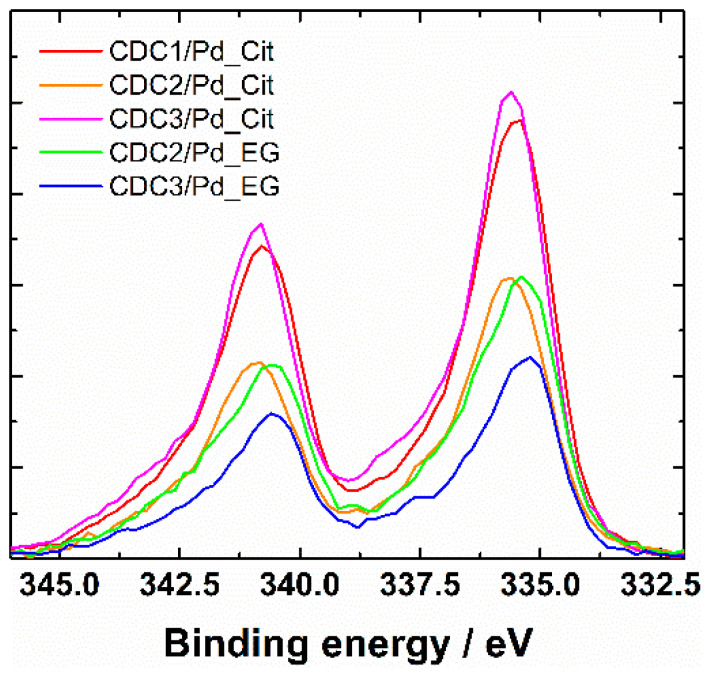
XPS Pd3d spectra of CDCx/Pd materials.

XRD patterns showed the presence of Pd_4_S in the case of CDC3/Pd_EG (reflections at 35°, 37.5°, and 42.7° can be attributed to Pd_4_S, Figure 3). All the samples show loosely connected layered carbon material with some graphitized carbon represented by (002) reflection at 26.4°. In the case of the citrate synthesis method, the palladium catalyst could be separated into two fractions, highly dispersed tiny crystallites or amorphous Pd and larger crystallites, listed in Table 1. Lattice constants (Table 1) observed for catalysts prepared by the citrate method are typical to that of Pd at 3.859 Å [48] for one of the phases; however, in the case of CDC1, palladium crystallite size is more variable. Two palladium structures were used to approximate its characterization. CDC2 and CDC3 had very finely dispersed Pd, which made determining lattice constant inaccurate, and thus only lattice constant for larger particles are provided. We can observe the differences in crystallite size distributions by comparing the XRD patterns and particle sizes counted from TEM images. Since the XRD method encompasses a more significant part of the sample, the average crystallite size for CDC3/Pd_EG is comparable to that of CDC2/Pd_EG, with N-doped CDC3 support providing a narrower PdNP size distribution. The difference in the TEM data could be due to larger Pd_4_S particles. CDCx/Pd-material XPS-survey spectra are shown in Figure S4, and Pd3d XPS spectra are provided in Figure 4 (with deconvolutions, Figure S5), and the presence of N-doping was also confirmed by XPS [51] (Figure S6 and Table S2). A slight difference is observed in the Pd3d XPS peaks between the EG and citrate synthesis methods on CDC2 and CDC3 substrates. The observation of the 3d_3/2_ peak at ca. 333.4 eV shows that different samples have different ratios of metallic palladium (at 335.4 eV) and Pd^2+^ (336.4 eV [52]). Palladium species and their distribution are provided in Table S3.

### 3.3. CV and CO-Stripping Measurements

**Figure 5:**
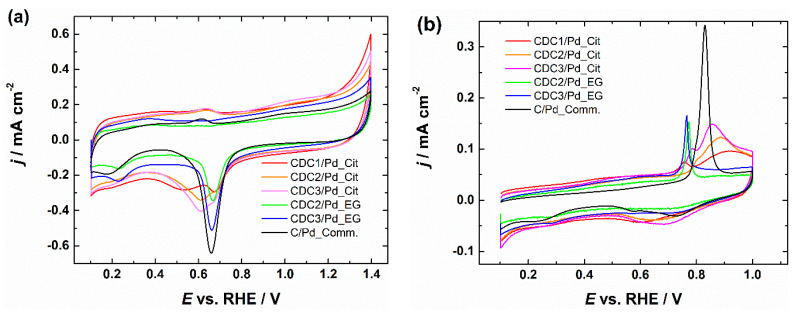
(**a**) CV curves of CDC/Pd catalysts in Ar-saturated 0.1 M KOH, *v* = 50 mV s^−1^. (**b**) Oxidation of pre-adsorbed CO on CDC/Pd catalysts, *v* = 20 mV s^−1^. Current densities are normalized to the geometric area of GC.

From CVs, we can see that the typical PdO reduction peak is broadened by a second peak in the case of the citrate synthesis method, which can be due to smaller Pd crystallites, as it has been shown that Pd oxide species are generally more stable on smaller particles, leading to a shift in the peak location [8]. This effect does not appear in the case of the EG synthesis method, as these smaller crystallites were observed during XRD only in the case of the citrate method. The CV profile of the CDCx/Pd_EG catalyst looks typical (Figure 5a) and is comparable to the CV of commercial Pd/C [53]. After the ORR measurement, we can observe the change in the CV shape in the case of the citrate synthesis method (Figures S7–S11), while the EG method yields a similar CV to the commercial Pd/C. Since the measurements are carried out in an alkaline solution and CO stripping should be able to clean the PdNP surface from adsorbed citrate if the particles were contaminated [54], it would be observable during the CV studies; however, no cleaning effect is observed in the hydrogen desorption area even when CO stripping was carried out in acidic conditions (Figures S12–S16). However, changes in the peak shape closer to that of the commercial Pd/C can be observed, which can be attributed to the low stability of these smaller crystallites (Figures S7–S16). The problem with these CVs is that the typical method of using charge integration under the PdO reduction peak for the electrochemical surface area (ESA) determination is disrupted in the case of materials synthesized using the citrate method. The shape of the CO-stripping curves further complicates this. At the same time, Pd NPs prepared by the citrate method gives the typical single peak for the CO oxidation profile similar to commercial Pd/C, albeit at a lower potential (Figure 5b). For the EG synthesis method, two peaks can be observed, one at around the same potential as for the CDCx/Pd_Cit material and the second broader peak stretching to higher potentials. We have previously observed similar CO-stripping behavior for Pd-based catalysts deposited on CDC and other highly porous carbon materials. The effect seems to be independent of N-doping [55,56]. One possible explanation for the tailing of the CO-stripping peak can be the particle size, as the tailing of CO-stripping peaks has been previously observed by comparing two commercial Pd/C materials with two different particle size distributions [57]. With the double peak, the first peak in the case of Pt has been previously attributed to agglomeration [54]; another explanation for the first one is that catalytically active OH formed on defect sites [58]. However, this further complicates the determination of ESA, as the second method typically used for this purpose is unsuitable for comparing these catalyst materials. The specific activity (SA) values summarized in Table 2 were obtained using the PdO reduction method for ESA determination in the case of the EG synthesis method and the CO oxidation method for the citrate synthesis method, as determining ESA based on H_udp_ in alkaline media on Pd electrodes is unreliable.

### 3.4. RDE Measurements

**Figure 6:**
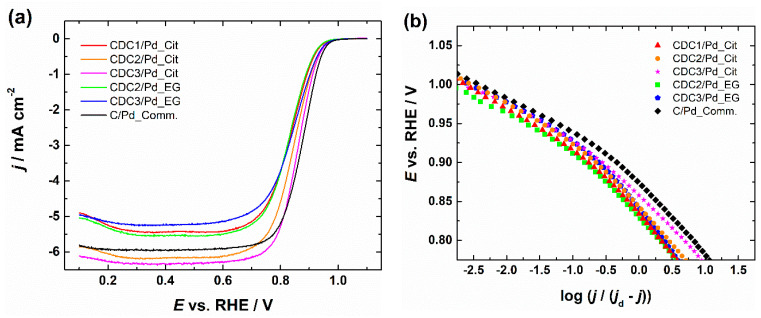
(**a**) Comparison of RDE polarization curves for oxygen reduction in O_2_-saturated 0.1 M KOH solution and (**b**) corresponding Tafel plots, *ω* = 1900 rpm, *v* = 10 mV s^−1^. Current densities are normalized to the geometric area of GC.

RDE polarization curves for ORR and the corresponding Koutecky–Levich plots are shown in Figures S17–S21, with insets showing the number of electrons transferred (*n*) calculated by Equation (S1) using constants [59,60]; all of the catalysts gave *n* value ~4, which is typical for a Pd catalyst. A comparison of catalyst materials in 0.1 M KOH solution at 1900 rpm can be seen in Figure 6. The ORR activity in terms of half-wave potentials (*E*_1/2_) of the catalysts prepared by the citrate method follows the order of CDC3/Pd > CDC2/Pd > CDC1/Pd, and the Pd catalyst supported on predominantly microporous CDC is performing significantly worse than those deposited on the other two CDC supports with mostly mesopores. Furthermore, CDC3/Pd material with N-doped support outperforms non-doped counterparts; a similar effect can also be observed for the EG synthesis method, as CDC3/Pd outperforms CDC2/Pd.

Equations (1) and (2) were used to calculate specific (SA) and mass activity (MA):

SA = *I_k_*/ESA (1)

MA = *I_k_*/*m*, (2)

where *I_k_* is the kinetic current at a specific potential, and ESA is the surface area of Pd determined by CO stripping or PdO reduction methods. *m* represents Pd mass obtained by MP-AES measurement.

Furthermore, we can see that the citrate synthesis method, which produced smaller PdNPs, is showing higher specific activity compared to EG synthesized counterparts as comparing the Pd particle sizes (average particle sizes for CDC2/Pd were 2.8 ± 0.5, 2.2 ± 0.3 nm and for CDC3/Pd 3.4 ± 1.6, 1.8 ± 0.4 nm for EG and citrate synthesis, respectively). However, since CDC2/Pd_EG particles were smaller than those of CDC3/Pd_EG, this would suggest that the presence of N-doping has a more substantial impact on the ORR activity than just decreasing Pd particle size, but this could also be due to the presence of Pd_4_S. Several researchers have discussed various effects of N-doping on the electrocatalytic activity of ORR. Gracia-Espino et al. suggested that N-doped graphene reduces the energy barrier of O_2_ dissociation [61]; however, a typical explanation is based on the increase in the number of anchoring sites [62,63]. Comparing the specific activities between the two synthesis methods is inaccurate as the electrochemical surface area is determined using different methods depending on the synthesis; for the CDC2- and CDC3-supported Pd catalysts, we can observe somewhat similar SA values, but compared to the PdNPs deposited on CDC1, which was predominantly microporous, a higher specific activity can be seen. This increase in SA can be attributed to the increased degree of agglomeration and growth of larger Pd crystallites that were determined by the XRD measurements, as it has been shown that larger Pd particles generally possess higher specific activity [8]. Mass activities provided in Table 2 show that different deposition methods resulted in similar MA values; furthermore, the effect of sulfur for the CDC3/Pd_EG is negligible. A comparison with the literature can be found in Table S4 [8,63,64,65,66,67,68,69,70].

Tafel slope values listed in Table 2 were determined in the potential range of 0.89–0.94 V. Tafel slope values are near −60 mV dec^−1^, which is typical for Pd/C catalysts [71,72]. A slightly higher Tafel slope of −69.2 mV dec^−1^ was calculated in the case of CDC3/Pd_EG material, which could be due to the presence of Pd_4_S as determined by XRD. A similar increase in Tafel slope was observed by Huang et al. in 0.5 M H_2_SO_4_ [73]. The −60 mV dec^−1^ slope is attributed to the oxide-covered Pd surface [74] with the rate-determining step for ORR being the transfer of the first electron to the O_2_ molecule [71,72]. The increase in Tafel slope has been previously attributed to the lowering of the oxide coverage on the Pd nanoparticles [75], which can be beneficial as PdO has been shown to promote the 2e^−^ ORR pathway [76].

## 4. Conclusions

Two synthesis approaches were used in this study to prepare Pd nanoparticles on the carbide-derived carbon supports, one of which was doped with nitrogen. No significant effect from N-doping was observed if SA was considered. However, smaller Pd particles were observed on the N-doped CDC, improving mass activity. An increase in the specific activity for ORR was noted for CDC1/Pd_Cit; however, this could be attributed to agglomeration and the formation of larger Pd particles, which was also observed by XRD. Both the CDC2 and CDC3 support materials had significant mesoporosity, which seems to facilitate better dispersion of the Pd nanoparticles. This is further improved in the case of N-doped support materials, resulting in higher ESA values as compared to the non-doped counterpart. While in the case of CDC3/Pd_Cit this could be attributed to smaller Pd particle size, in the case of CDC3/Pd_EG the particles were larger compared to the CDC2/Pd_EG material, which could suggest a lower degree of agglomeration of Pd nanoparticles in the N-doped CDC material.

## Figures and Tables

**Table 1:** Physical parameters of CDCx/Pd catalysts.

Catalyst	Particle Size (nm)	Pd LVol-FWHM ** (nm)	Lattice Parameter (Å)	Pd (wt%)
CDC1/Pd_Cit	2.1 ± 0.5	9.80 ± 0.26/1.21 ± 0.01	3.936/3.896	15.0
CDC2/Pd_Cit	2.2 ± 0.5	5.57 ± 0.89/0.60 ± 0.01	3.898	25.5
CDC3/Pd_Cit	1.8 ± 0.4	2.14 ± 0.08/0.50 ± 0.01	3.897	22.6
CDC2/Pd_EG	2.8 ± 0.7	2.09 ± 1.31	3.903	14.8
CDC3/Pd_EG	3.5 ± 1.4	2.18 ± 0.03/7.00 ± 1.45 *	3.893/5.1147 *	16.8

* Pd_4_S. ** volume-weighted mean crystallite size.

**Table 2:** Kinetic parameters of the prepared Pd-based catalysts for ORR in 0.1 M KOH solution.

Catalyst	ESA (cm^2^)	*E*_1/2_ (V)	Tafel Slope (mV dec^−1^) *	SA at 0.9 V (mA cm^−2^)	MA at 0.9 V (A g^−1^)
CDC1/Pd_Cit	0.206	0.834	−61	0.684	341
CDC2/Pd_Cit	0.307	0.844	−66	0.624	289
CDC3/Pd_Cit	0.392	0.858	−61	0.584	414
CDC2/Pd_EG	0.252	0.830	−60	0.512	276
CDC3/Pd_EG	0.356	0.842	−69	0.490	411
C/Pd_Comm	0.589	0.874	−60	0.487	670

* Determined in the potential range of 0.89–0.94 V.

## Data Availability

Dataset available on request from the authors.

## Supplementary Materials

The following supporting information can be downloaded at: https://www.mdpi.com/article/10.3390/nano14120994/s1, CDC_Pd_SI.docx. Table S1. Texture characteristics of CDC materials measured using standard methodology [1]. Table S2. Various surface species of the CDC3 support material determined by XPS. Table S3. Pd species in the catalyst materials determined by XPS. Table S4. Comparison with various Pd/C materials from literature. Figure S1. PSD graph of CDC materials. Figure S2. Lattice fringes for a) CDC1/Pd_Cit, b) CDC2/Pd_Cit, c) CDC3/Pd_Cit, d) CDC2/Pd_EG, and e) CDC3/Pd_EG samples. Figure S3. Pd particle size distribution for catalyst materials. Figure S4. Cascade of survey spectra for CDCx/Pd catalysts. Figure S5. Deconvolution of the Pd3d peak for a) CDC1/Pd_Cit, b) CDC2/Pd_Cit, c) CDC3/Pd_Cit, d) CDC2/Pd_EG, and e) CDC3/Pd_EG samples. Figure S6. (a) C1s, (b) O1s, and (c) N1s XPS spectra for CDC3 sample. Figure S7. CV curves of CDC1/Pd_Cit catalyst in Ar-saturated 0.1 M KOH solution, *v* = 50 mV s^−1^. Figure S8. CV curves of CDC2/Pd_Cit catalyst in Ar-saturated 0.1 M KOH solution, *v* = 50 mV s^−1^. Figure S9. CV curves of CDC3/Pd_Cit catalyst in Ar-saturated 0.1 M KOH solution, *v* = 50 mV s^−1^. Figure S10. CV curves of CDC2/Pd_EG catalyst in Ar-saturated 0.1 M KOH solution, *v* = 50 mV s^−1^. Figure S11. CV curves of CDC3/Pd_EG catalyst in Ar-saturated 0.1 M KOH solution, *v* = 50 mV s^−1^. Figure S12. CV curves of CDC1/Pd_Cit catalyst in Ar-saturated 0.5 M H_2_SO_4_ solution, *v* = 50 mV s^−1^. Figure S13. CV curves of CDC2/Pd_Cit catalyst in Ar-saturated 0.5 M H_2_SO_4_ solution, *v* = 50 mV s^−1^. Figure S14. CV curves of CDC3/Pd_Cit catalyst in Ar-saturated 0.5 M H_2_SO_4_ solution, *v* = 50 mV s^−1^. Figure S15. CV curves of CDC2/Pd_EG catalyst in Ar-saturated 0.5 M H_2_SO_4_ solution, *v* = 50 mV s^−1^. Figure S16. CV curves of CDC3/Pd_EG catalyst in Ar-saturated 0.5 M H_2_SO_4_ solution, *v* = 50 mV s^−1^. Figure S17. a) RDE results of CDC1/Pd_Cit in O_2_-saturated 0.1 M KOH and b) corresponding K–L plots, inset shows the potential dependence of n. Figure S18. a) RDE results of CDC2/Pd_Cit in O_2_-saturated 0.1 M KOH and b) corresponding K–L plots, inset shows the potential dependence of n. Figure S19. a) RDE results of CDC3/Pd_Cit in O_2_-saturated 0.1 M KOH and b) corresponding K–L plots, inset shows the potential dependence of n. Figure S20. a) RDE results of CDC2/Pd_EG in O_2_-saturated 0.1 M KOH and b) corresponding K–L plots, inset shows the potential dependence of n. Figure S21. a) RDE results of CDC3/Pd_EG in O_2_-saturated 0.1 M KOH and b) corresponding K–L plots, inset shows the potential dependence of n. References [8,45,51,59,60,63,64,65,66,67,68,69,70] are cited in the Supplementary Materials.
