# Supplementary material for: One-Pot Synthesis of Pd Nanoparticles Supported on Carbide-Derived Carbon for Oxygen Reduction Reaction
Source: Nanomaterials (Basel). 2024 Jun 7;14(12):994. doi: 10.3390/nano14120994 (PMC11206402; doi:10.3390/nano14120994)
Supplement: Supplementary file 1 [file nanomaterials-14-00994-s001.zip › nanomaterials-3014932-supplementary.pdf]

Supplementary Materials

One-pot synthesis of Pd nanoparticles supported on carbide-derived carbon for oxygen reduction reaction

Madis Lüsi[a], Heiki Erikson[a], Maike Käärik[a] Helle-Mai Piirsoo[b], Jaan Aruväli[c], Arvo Kikas[b], Vambola Kisand[b], Jaan Leis[a], Kaupo Kukli[b], Kaido Tammeveski[a,*]

[a]*Institute of Chemistry, University of Tartu, Ravila 14a, 50411 Tartu, Estonia*
[b]*Institute of Physics, University of Tartu, W. Ostwald Str. 1, 50411 Tartu, Estonia*
[c]*Institute of Ecology and Earth Sciences, University of Tartu, Vanemuise 46, 51014 Tartu, Estonia*

*Corresponding author. Tel.: +372 7375168; E-mail: kaido.tammeveski@ut.ee (K. Tammeveski)

Pore size distribution (PSD) of CDC materials

Table S1. Texture characteristics of CDC materials measured using standard methodology [1].

Sample	Precursor	SSA_{BET} ($m^2\ g^{-1}$)	S_{dft} ($m^2\ g^{-1}$)	V_{mic} ($cm^3\ g^{-1}$)	V_{tot} ($cm^3\ g^{-1}$)	S_{dft} mic ($m^2\ g^{-1}$)	S_{dft} meso ($m^2\ g^{-1}$)
CDC-1	TiC-PT with steam	2080	1803	0.84	1.1	1701	102
CDC-2	B_4C	1588	1386	0.55	1.13	1076	1385
CDC-3	TiCN 50/50	838	828	0.26	0.94	593	828

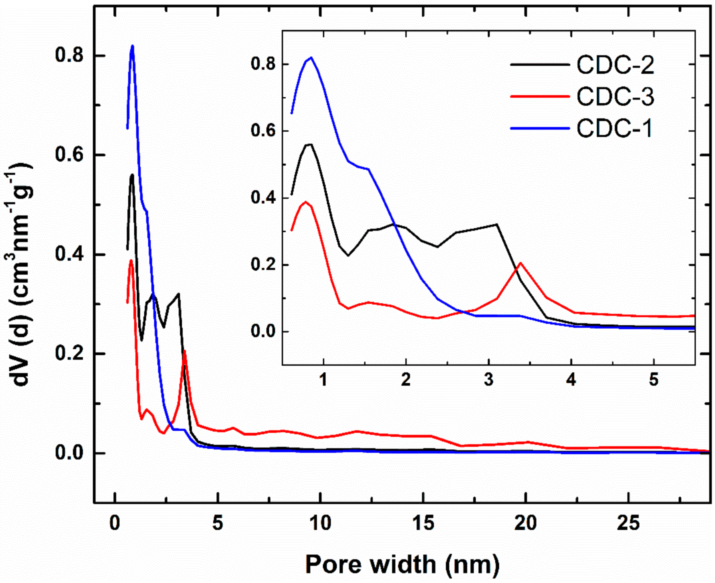

Figure S1. PSD graph of CDC materials.

STEM analysis for CDCx/Pd materials

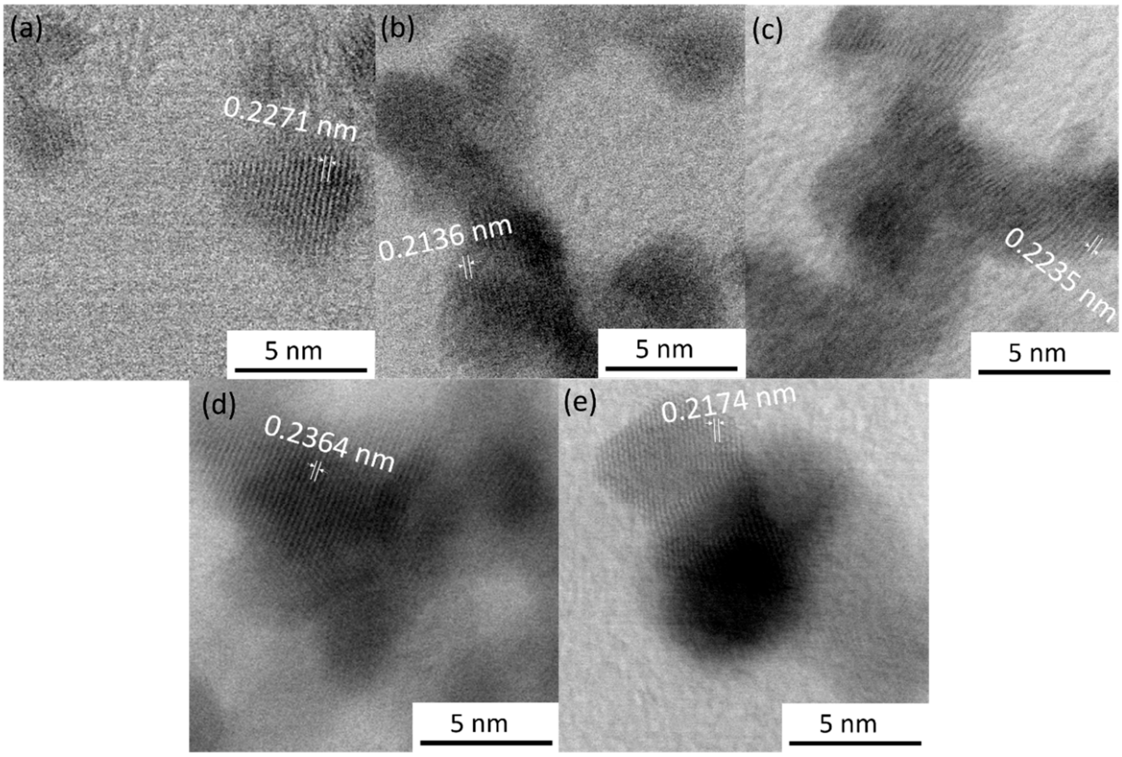

Figure S2. Lattice fringes for a) CDC1/Pd_Cit, b) CDC2/Pd_Cit, c) CDC3/Pd_Cit, d) CDC2/Pd_EG, and e) CDC3/Pd_EG samples.

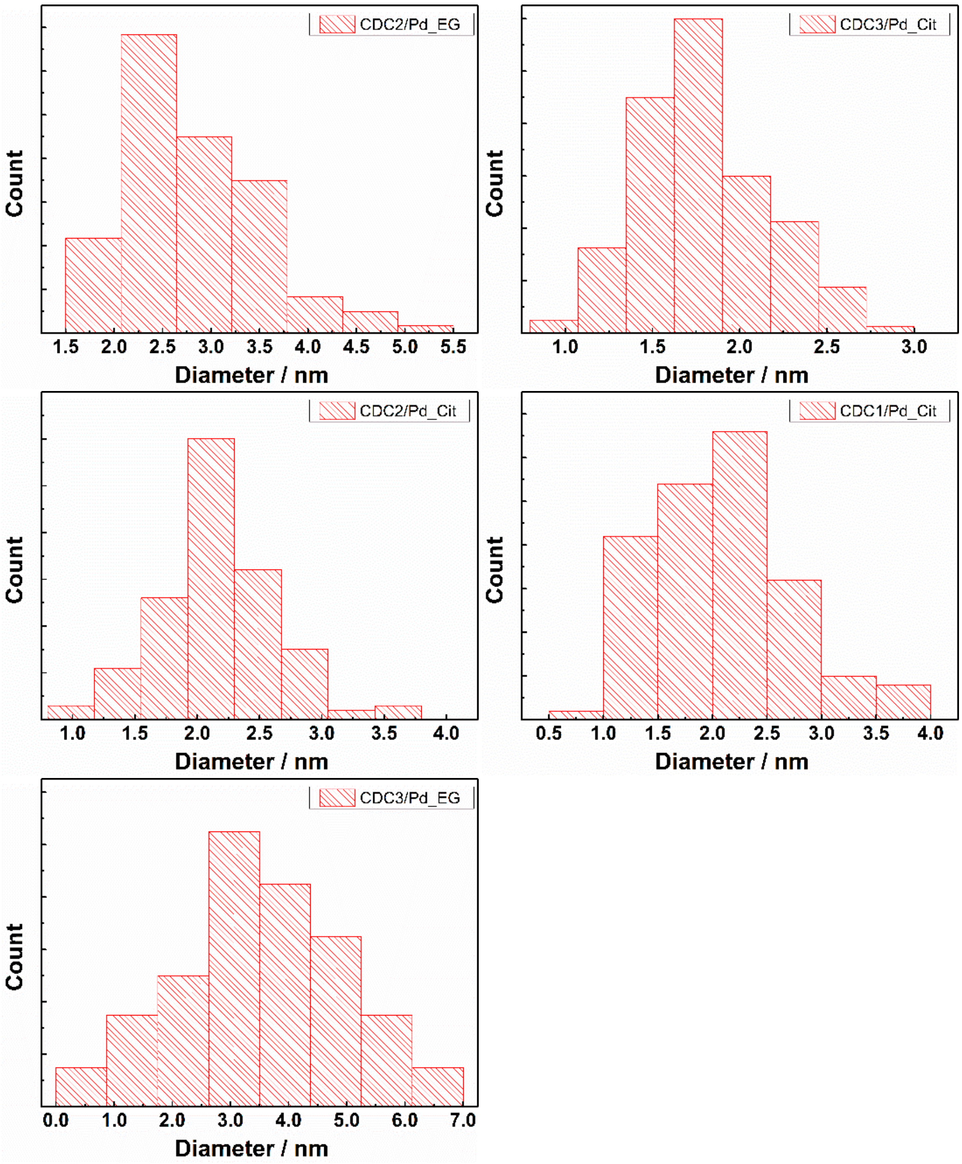

Figure S3. Pd particle size distribution for catalyst materials.

XPS analysis

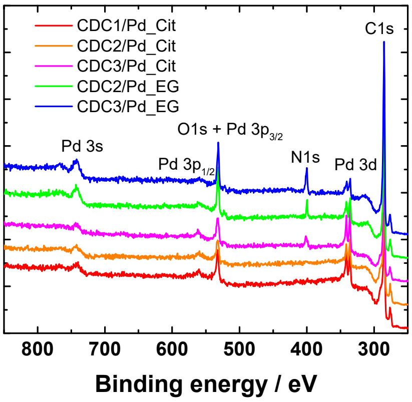

Figure S4 Cascade of survey spectra for CDCx/Pd catalysts.

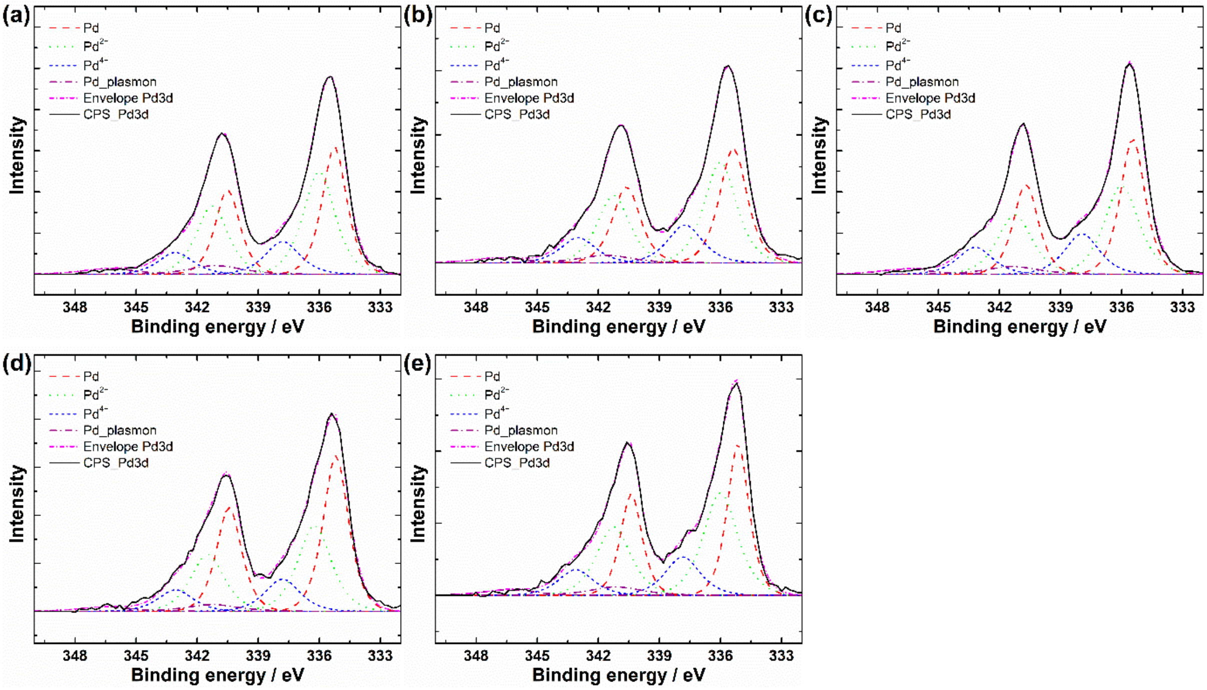

Figure S5. Deconvolution of the Pd3d peak for a) CDC1/Pd_Cit, b) CDC2/Pd_Cit, c) CDC3/Pd_Cit, d) CDC2/Pd_EG, and e) CDC3/Pd_EG samples.

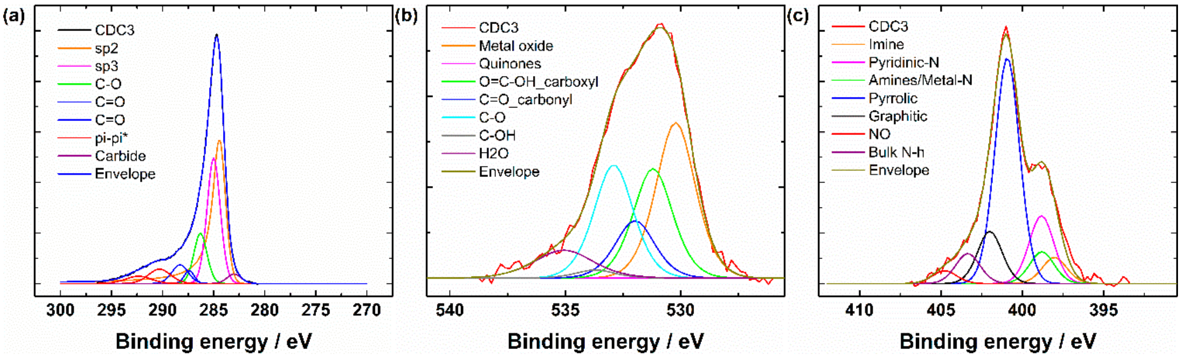

Figure S6. (a) C1s, (b) O1s, and (c) N1s XPS spectra for CDC3 sample.

Following peaks are introduced for carbon [2]: carbide at 283.0 eV, sp^2-C at 284.5 eV, sp^3-C at 285.0 eV, C-O at 286.3 eV, C=O at 287.4 eV, and O-C=O at 288.3 eV. The peaks with higher binding energy at 290.3 and 294.3 eV are attributed to the (pii)-> (pii)* shake up excitations.

Table S2. Various surface species of the CDC3 support material determined by XPS.

C1s	BE (eV)	at%	O1s	BE (eV)	at%	N1s	BE (eV)	at%
sp^2	284.5	44.89	metal oxide	530.2	0.92	Imine	398.0	0.34
sp^3	285	22.54	quinones	530.2	0	Pyridinic-N	398.8	0.89
C-O	286.3	8.99	O=C-OH carboxyl	531.2	0.65	Amines/ Metal-Nx	398.8	0.42
O-C=O	288.3	4.18	C=O carbonyl	532.0	0.34	Pyrrolic	400.9	2.96
C=O	287.4	1.74	C-O	532.9	0.67	Graphitic	402.0	0.69
pi-pi*	290.3	4.41	C-OH	533.7	0.05	NO	404.8	0.17

pi-pi*	292.4	2.62	Water, chemisorbed O	535.1	0.25	bulk N-h	403.3	0.39
carbide	283	1.86						

Table S3. Pd species in the catalyst materials determined by XPS.

Sample	PdO (at%)	PdO_2 (at%)	Pd (at%)	Pd plasmon (at%)
CDC1/Pd_Cit	0.8	0.28	0.77	0.12
CDC2/Pd_Cit	0.49	0.2	0.47	0.06
CDC3/Pd_Cit	0.97	0.45	1.05	0.13
CDC2/Pd_EG	0.45	0.17	0.6	0.05
CDC3/Pd_EG	0.33	0.1	0.3	0.05

Cyclic voltammetry

CV curves measured before, after CO-stripping, and after ORR experiments were named CV1, CV2, and CV3, respectively.

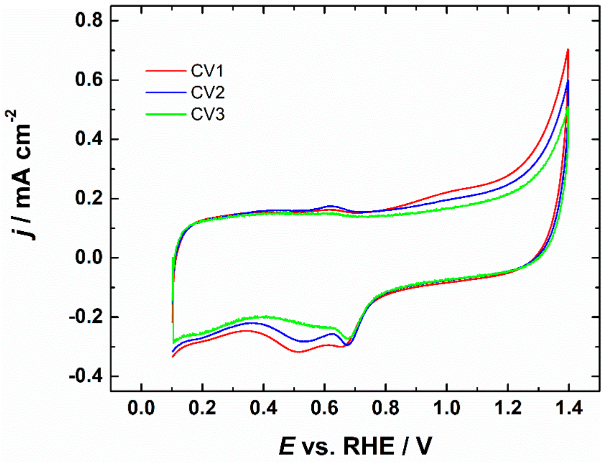

Figure S7. CV curves of CDC1/Pd_Cit catalyst in Ar-saturated 0.1 M KOH solution, v = 50 mV s^{-1}.

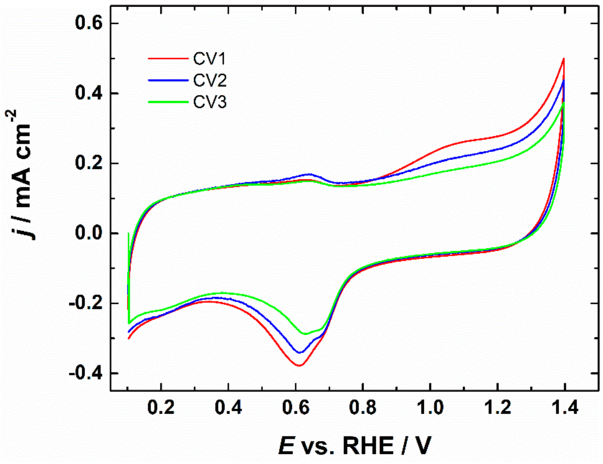

Figure S8. CV curves of CDC2/Pd_Cit catalyst in Ar-saturated 0.1 M KOH solution, v = 50 mV s^{-1}.

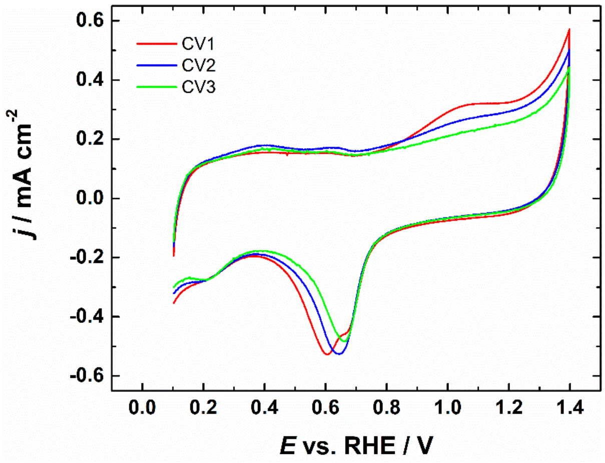

Figure S9. CV curves of CDC3/Pd_Cit catalyst in Ar-saturated 0.1 M KOH solution, $v = 50$ mV s^{-1}.

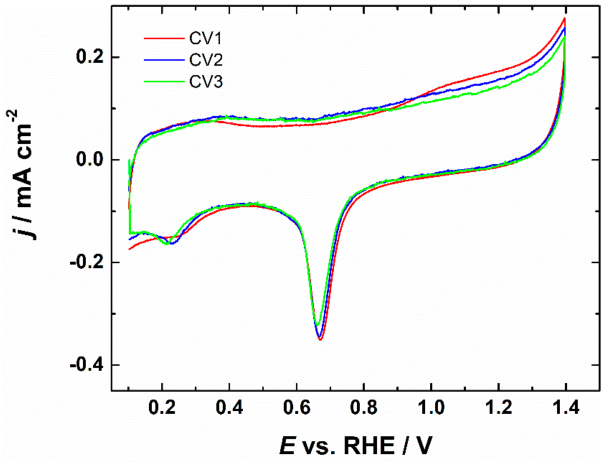

Figure S10. CV curves of CDC2/Pd_EG catalyst in Ar-saturated 0.1 M KOH solution, $v = 50$ mV s^{-1}.

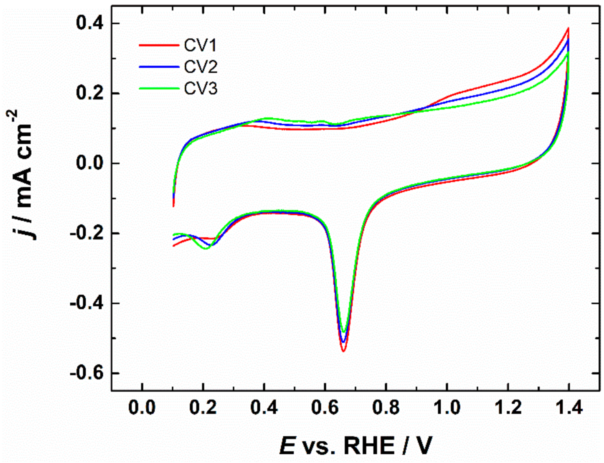

Figure S11. CV curves of CDC3/Pd_EG catalyst in Ar-saturated 0.1 M KOH solution, $v = 50$ mV s^{-1}.

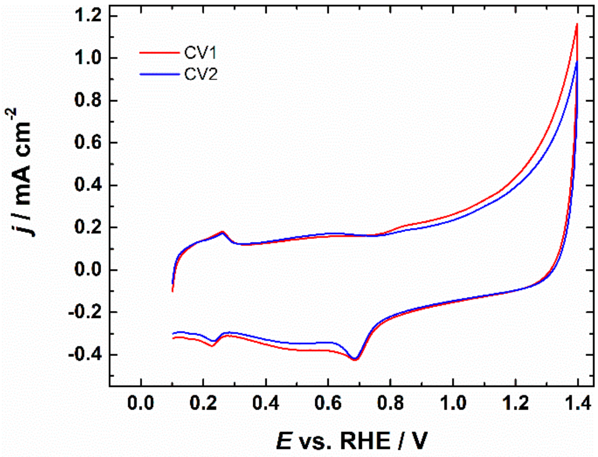

Figure S12. CV curves of CDC1/Pd_Cit catalyst in Ar-saturated 0.5 M H_2SO_4 solution, $v = 50$ mV s^{-1}

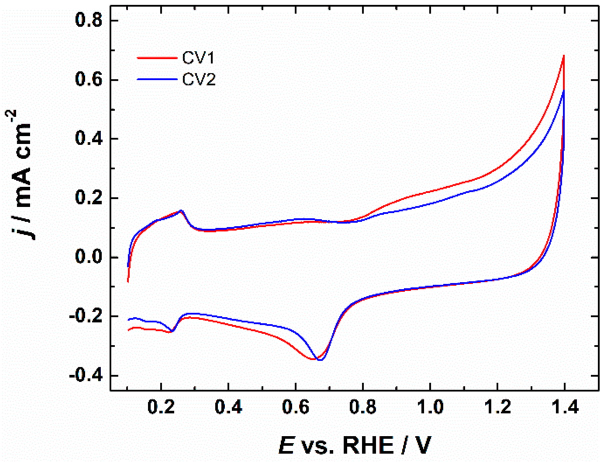

Figure S13. CV curves of CDC2/Pd_Cit catalyst in Ar-saturated 0.5 M H_2SO_4 solution, $v = 50$ mV s^{-1}

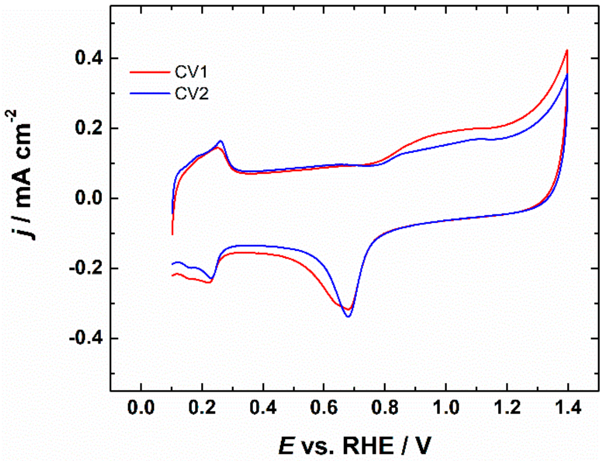

Figure S14. CV curves of CDC3/Pd_Cit catalyst in Ar-saturated 0.5 M H_2SO_4 solution, $v = 50$ mV s^{-1}

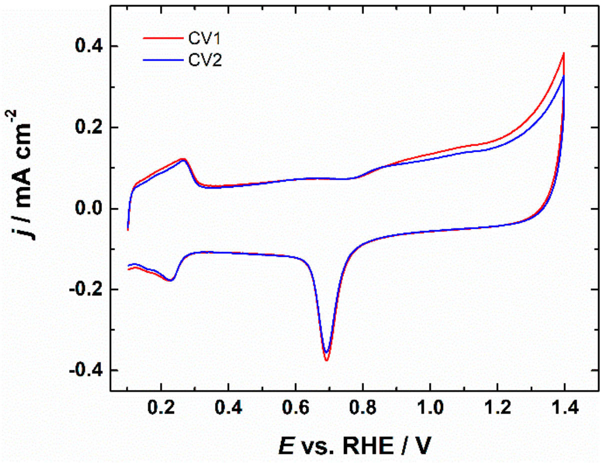

Figure S15. CV curves of CDC2/Pd_EG catalyst in Ar-saturated 0.5 M H_2SO_4 solution, $v = 50$ mV s^{-1}

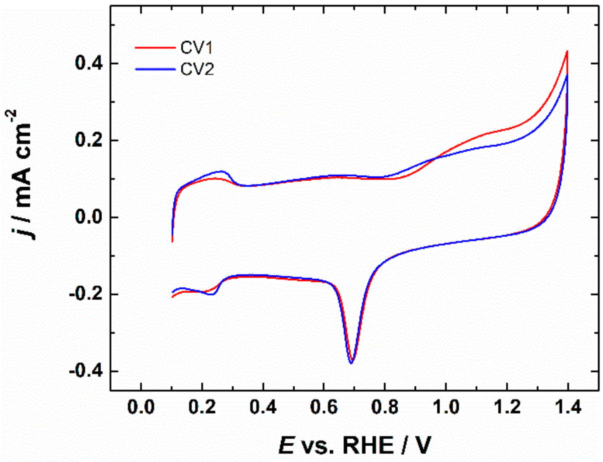

Figure S16. CV curves of CDC3/Pd_EG catalyst in Ar-saturated 0.5 M H_2SO_4 solution, $v = 50$ mV s^{-1}

ORR studies

RDE polarization data was analyzed using the Koutecky-Levich (K-L) equation:

$$\frac{1}{j} = \frac{1}{j_k} + \frac{1}{j_d} = -\frac{1}{nFkC^b_{O_2}} - \frac{1}{0.62nFD^{2/3}_{O_2}\nu^{-1/6}C^b_{O_2}\omega^{1/2}} \quad \text{(S1)}$$

where j is the overall O_2 reduction current density, and j_k represents kinetic and j_d diffusion-limited current densities. k is the electrochemical rate constant for O_2 reduction (cm s^{-1}), n is the number of electrons transferred per O_2 molecule, F is the Faraday constant (96,485 C mol^{-1}), and ω is the rotation rate of the electrode (rad s^{-1}). For O_2-saturated 0.1 M KOH solution, these values were used: kinematic viscosity ($\nu = 0.01$ cm^2 s^{-1} [3]), concentration of O_2 ($C^b_{O2} = 1.2\times10^{-6}$ mol cm^{-3} [4]), diffusion coefficient of O_2 ($D_{O2} = 1.9\times10^{-5}$ cm^2 s^{-1} [4]).

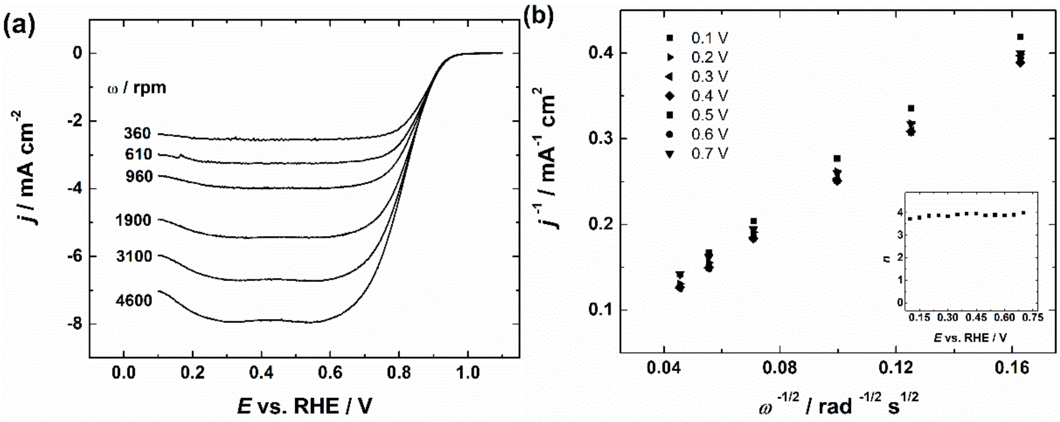

Figure S17. a) RDE results of CDC1/Pd_Cit in O_2-saturated 0.1 M KOH and b) corresponding K–L plots, inset shows the potential dependence of n.

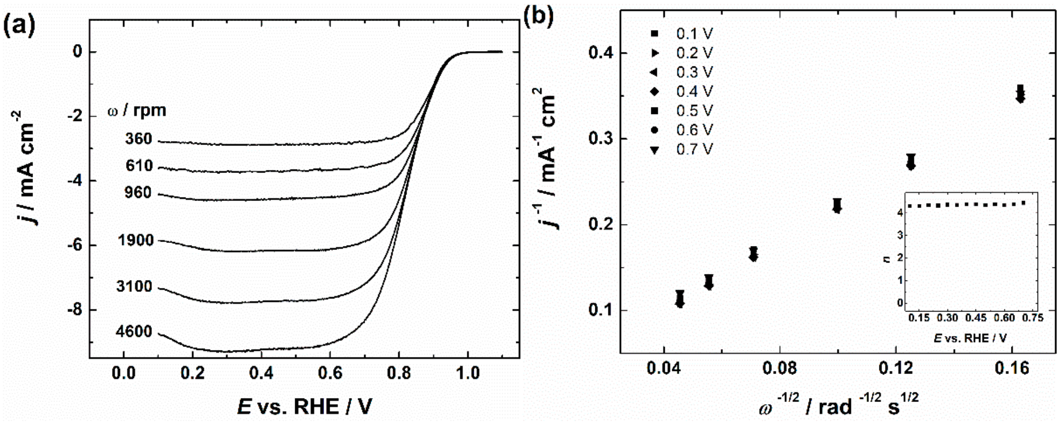

Figure S18. a) RDE results of CDC2/Pd_Cit in O_2-saturated 0.1 M KOH and b) corresponding K–L plots, inset shows the potential dependence of n.

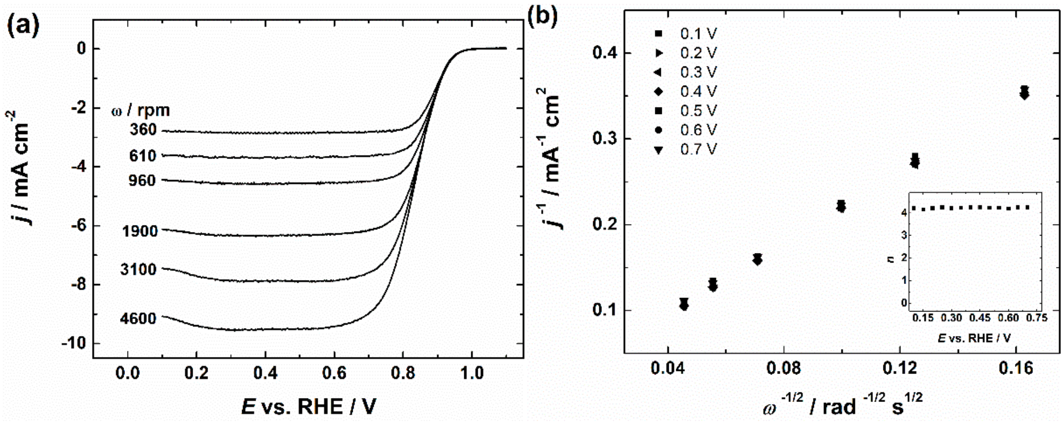

Figure S19. a) RDE results of CDC3/Pd_Cit in O_2-saturated 0.1 M KOH and b) corresponding K–L plots, inset shows the potential dependence of n.

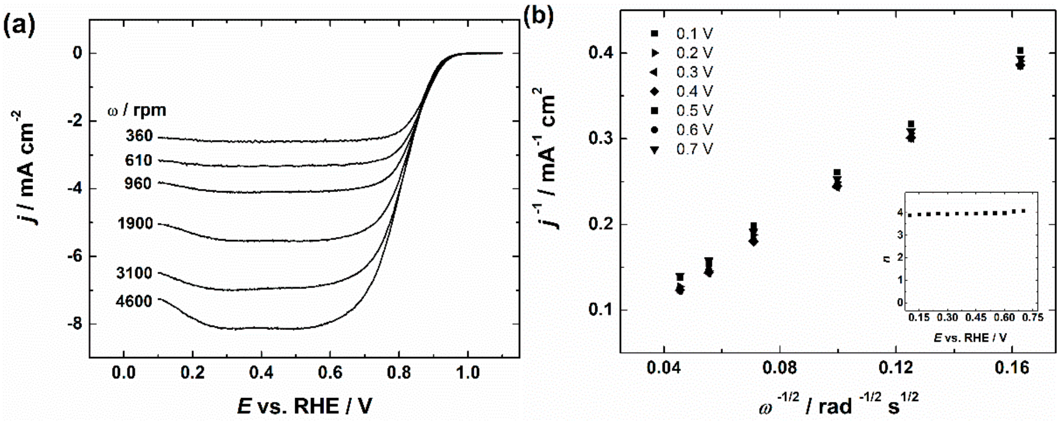

Figure S20. a) RDE results of CDC2/Pd_EG in O_2-saturated 0.1 M KOH and b) corresponding K–L plots, inset shows the potential dependence of n.

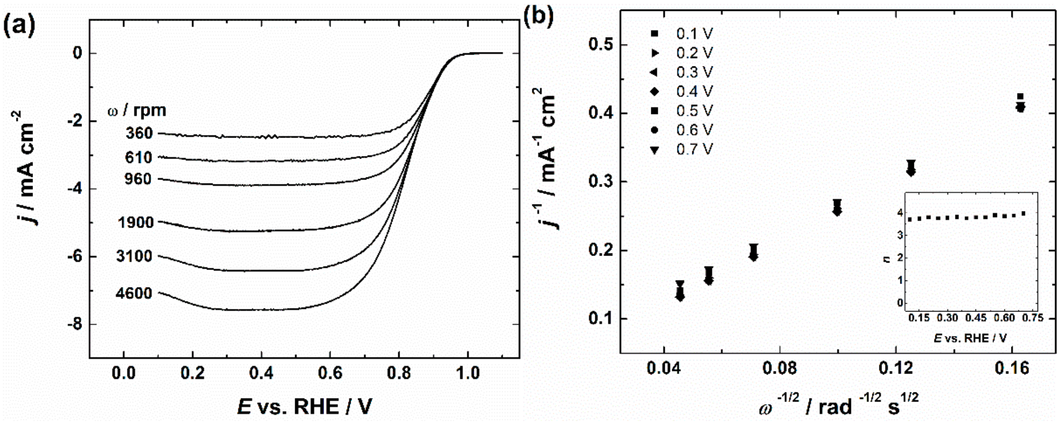

Figure S21. a) RDE results of CDC3/Pd_EG in O_2-saturated 0.1 M KOH and b) corresponding K–L plots, inset shows the potential dependence of n.

Table S4. Comparison with various Pd/C materials from literature.

Catalyst	$E_{1/2}$ (V)	SA at 0.9 V (mA cm^{-2})	MA at 0.9 V (A g^{-1})	Reference
CDC2/Pd_Cit	0.844	0.624	289	This work
CDC2/Pd_EG	0.830	0.512	276	This work
h-BN/C/Pd:17	0.91	0.86	250	[5]
Pd/C	0.866	0.130	98	[6]
Pd-B/C	0.883	0.170	145	[6]
Pd/CSN	N/A	0.114	286	[7]
Pd10/nCNF	0.769	N/A	174	[8]
Pd-HCS-700	0.802	N/A	60	[9]
Pd/C	0.868	~0.92	~43	[10]
Pd/N-HsGY	0.849	~0.33[1]	N/A	[11]
Pd MSs	N/A	~1.25	~440	[12]
Pd/C-400	N/A	~0.20	~104	[13]

[1]Taken from a Figure.

References

[1] M. Käärik, M. Arulepp, M. Käärik, U. Maran, J. Leis, Characterization and prediction of double-layer capacitance of nanoporous carbon materials using the Quantitative nano-Structure-Property Relationship approach based on experimentally determined porosity descriptors, Carbon 158 (2020) 494-504.
[2] M. Brzhezinskaya, E.A. Belenkov, V.A. Greshnyakov, G.E. Yalovega, I.O. Bashkin, New aspects in the study of carbon-hydrogen interaction in hydrogenated carbon nanotubes for energy storage applications, Journal of Alloys and Compounds 792 (2019) 713-720.
[3] D.R. Lide, CRC handbook of chemistry, physics, CRC Press, Boca Raton, 2001.
[4] R.E. Davis, G.L. Horvath, C.W. Tobias, The solubility and diffusion coefficient of oxygen in potassium hydroxide solutions, Electrochimica Acta 12(3) (1967) 287-297.
[5] Y. Chen, J. Cai, P. Li, G. Zhao, G. Wang, Y. Jiang, J. Chen, S.X. Dou, H. Pan, W. Sun, Hexagonal Boron Nitride as a Multifunctional Support for Engineering Efficient Electrocatalysts toward the Oxygen Reduction Reaction, Nano Letters 20(9) (2020) 6807-6814.
[6] M. Wang, X. Qin, K. Jiang, Y. Dong, M. Shao, W.-B. Cai, Electrocatalytic Activities of Oxygen Reduction Reaction on Pd/C and Pd–B/C Catalysts, The Journal of Physical Chemistry C 121(6) (2017) 3416-3423.
[7] W. Yan, Z. Tang, L. Li, L. Wang, H. Yang, Q. Wang, W. Wu, S. Chen, Ultrasmall Palladium Nanoclusters Encapsulated in Porous Carbon Nanosheets for Oxygen Electroreduction in Alkaline Media, ChemElectroChem 4(6) (2017) 1349-1355.
[8] M.A. Khalily, B. Patil, E. Yilmaz, T. Uyar, Atomic Layer Deposition of Pd Nanoparticles on N-Doped Electrospun Carbon Nanofibers: Optimization of ORR Activity of Pd-Based Nanocatalysts by Tuning Their Nanoparticle Size and Loading, ChemNanoMat 5(12) (2019) 1540-1546.
[9] X. Wang, Z. Chen, S. Chen, H. Wang, M. Huang, Nitrogen and Oxygen Co-Doping Assisted Synthesis of Highly Dispersed Pd Nanoparticles on Hollow Carbon Spheres as Efficient Electrocatalysts for Oxygen Reduction Reaction, Chemistry – A European Journal 26(55) (2020) 12589-12595.
[10] Y. Yang, G. Chen, R. Zeng, A.M. Villarino, F.J. DiSalvo, R.B. van Dover, H.D. Abruña, Combinatorial Studies of Palladium-Based Oxygen Reduction Electrocatalysts for Alkaline Fuel Cells, Journal of the American Chemical Society 142(8) (2020) 3980-3988.
[11] W. Si, Z. Yang, X. Hu, Q. Lv, X. Li, F. Zhao, J. He, C. Huang, Preparation of zero valence Pd nanoparticles with ultra-efficient electrocatalytic activity for ORR, Journal of Materials Chemistry A 9(25) (2021) 14507-14514.
[12] H. Lv, D. Xu, L. Sun, J. Henzie, S.L. Suib, Y. Yamauchi, B. Liu, Ternary Palladium–Boron–Phosphorus Alloy Mesoporous Nanospheres for Highly Efficient Electrocatalysis, ACS Nano 13(10) (2019) 12052-12061.
[13] L. Jiang, A. Hsu, D. Chu, R. Chen, Size-Dependent Activity of Palladium Nanoparticles for Oxygen Electroreduction in Alkaline Solutions, Journal of the Electrochemical Society 156(5) (2009) B643-B649.
